# Y‐box binding protein 1 stabilizes EP300 mRNA and promotes forkhead box C1 H3K27Ac to aggravate chondrocyte injury in osteoarthritis

**DOI:** 10.1002/ccs3.70028

**Published:** 2025-07-23

**Authors:** Jingyi Li, Gang Zhou, Te Chen, Qiao Lin, Qiupeng Yang

**Affiliations:** ^1^ Joint Surgical Department Hainan General Hospital Haikou Hainan Province China

**Keywords:** chondrocyte injury, EP300, FOXC1, osteoarthritis, YBX1

## Abstract

Chondrocyte abnormalities play an important role in osteoarthritis (OA), and forkhead box C1 (FOXC1) expression is related to OA progression. Nonetheless, the molecular mechanisms underlying the action of FOXC1 in chondrocytes remain unclear. Rats were subjected to anterior cruciate ligament transection (ACLT) to establish an in vivo OA model, and chondrocytes were subjected to interleukin (IL)‐1β to establish an in vitro OA model. Pathological changes in rat cartilage tissues were evaluated using hematoxylin–eosin and safranin O staining. H3K27Ac enrichment in the FOXC1 promoter was analyzed using chromatin immunoprecipitation. Interactions between EP300 and Y‐box binding protein 1 (YBX1) were validated using RNA immunoprecipitation and RNA pull‐down assay. The expression of YBX1, EP300, and FOXC1 was elevated in ACLT rats and IL‐1β‐induced chondrocytes. FOXC1 knockdown inhibited apoptosis and inflammatory response in IL‐1β‐induced chondrocytes. EP300 bound to FOXC1 promoter and promoted H3K27Ac enrichment in the FOXC1 promoter. Additionally, YBX1 bound to EP300 mRNA and enhanced EP300 mRNA stability. YBX1 overexpression promoted cell apoptosis and inflammation of IL‐1β‐induced chondrocytes, but was reversed by FOXC1 downregulation. YBX1 enhances EP300 mRNA stability and elevates FOXC1 expression by mediating FOXC1 H3K27Ac to promote IL‐1β‐induced chondrocyte apoptosis and inflammation, thereby exacerbating chondrocyte injury in OA.

## Graphical abstract

In this study, YBX1 binds to EP300 mRNA and enhances its stability and expression. Subsequently, EP300 binds to the FOXC1 promoter and promotes the enrichment of H3K27Ac at the FOXC1 promoter to elevate FOXC1 expression, ultimately regulating IL‐1β‐induced chondrocyte damage and promoting the progression of osteoarthritis.

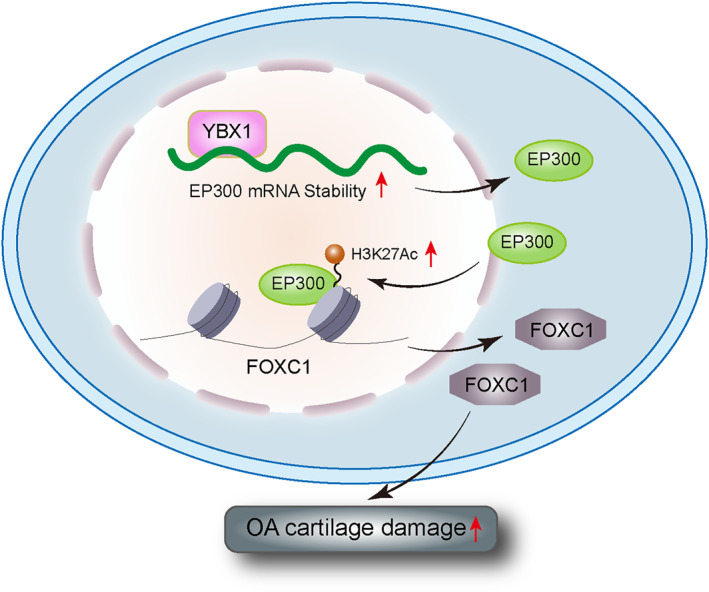

## 1 INTRODUCTION

Osteoarthritis (OA) is a prevalent, chronic, and degenerative joint disease that typically develops with age. Its hallmark symptoms include joint stiffness, pain, and swelling, all of which can considerably impair the quality of life of affected individuals.^1^ Current therapies for OA primarily include drug therapy and surgical intervention. In the early stages of OA, drug therapy is often the first‐line treatment, and nonsteroidal anti‐inflammatory drugs are commonly used to alleviate pain and reduce inflammation. For end‐stage OA, the most common treatment is surgical intervention such as joint replacement.^2^ However, these drugs are associated with adverse reactions and limited effects, whereas surgery carries inherent risks and potential complications.^3^, ^4^ Previous studies have confirmed that articular cartilage degeneration is a central event in the pathogenesis of OA. During OA progression, chondrocyte differentiation/death and cartilage matrix synthesis/degradation are imbalanced. Numerous factors such as joint inflammation and oxidative stress induce excessive chondrocyte death, resulting in cartilage matrix degradation and subsequent loss of joint function.^5^ Therefore, exploring the pathogenesis of OA from the perspective of chondrocytes and identifying safer, more effective, and reliable therapeutic targets are important.

Forkhead box C1 (FOXC1), a member of the FOX transcription factor family, binds to target genes via its unique DNA‐binding domain, the forkhead region, to initiate transcription and protein expression.^6^ Previous studies have extensively investigated FOXC1 in inflammation‐related diseases such as ischemic stroke, ulcerative colitis, and rheumatoid arthritis.^7^, ^8^, ^9^ Moreover, Qi Fei et al. conducted an analysis of the GEO database and identified FOXC1 as a pivotal gene in OA.^10^ Another report indicated that FOXC1 was highly expressed in OA and promoted inflammation in synovial fibroblasts.^11^ Jun Wang et al. revealed that FoxC1 was upregulated in the synovial membranes and synovial fibroblasts in OA, where it promotes OA progression.^12^ Collectively, these findings suggest that FOXC1 plays a pivotal role in OA progression. Nonetheless, the molecular mechanisms underlying FOXC1‐mediated OA development and whether FOXC1 regulates chondrocyte function remain unclear.

Histone acetylation modification, an extensively studied form of epigenetic inheritance, plays a crucial role in the progression of multiple diseases.^13^, ^14^ Emerging evidence suggests that histone acetylation is significantly involved in the pathogenesis of OA.^15^ In breast cancer, PcG proteins promote FOXC1 expression by enhancing H3/H4 acetylation of the FOXC1 promoter.^16^ It is worth noting that the University of California, Santa Cruz (UCSC; https://genome.ucsc.edu), using the ENCODE database (https://www.encodeproject.org/), documented histone H3 lysine 27 acetylation (H3K27Ac) in the promoter region of FOXC1, indicating that FOXC1 expression may be regulated by H3K27Ac. E1A binding protein p300 (EP300), a histone acetyltransferase, is responsible for catalyzing H3K27Ac.^17^ EP300 has been implicated in cell proliferation, differentiation, apoptosis, and epigenetic modifications via the acetylation of target proteins and transcription factors.^18^, ^19^ Growing evidence has linked EP300 dysregulation to various diseases such as diabetes, myocardial hypertrophy, and OA.^20^, ^21^, ^22^ Velasco et al. suggested that EP300 expression is abnormally upregulated in knee OA.^23^ Furthermore, we discovered an interaction between EP300 and FOXC1; however, their connection with OA has not been reported yet.

The RNA‐binding protein (RBP) Y‐box binding protein 1 (YBX1) is an important regulatory factor in transcription and translation, participating in RNA selective cleavage, RNA stability, and RNA degradation by binding to target mRNAs.^24^, ^25^ YBX1 has been shown by previous research to participate in multiple biological processes, including mitochondrial function, inflammation, and oxidative stress.^26^ Abnormal YBX1 expression has been implicated in OA progression. For instance, YBX1, upregulated in human OA tissues, is regulated by miR‐379‐5p, and suppresses chondrocyte proliferation in OA by influencing the PI3K/Akt pathway.^27^ In addition, previous studies have revealed that YBX1, an RBP, enhances the stability of Pink1 and Prkn mRNA.^28^ We predicted the binding site between YBX1 and EP300 mRNA using the starBase database (http://starbase.sysu.edu.cn/). We hypothesized that YBX1 affects chondrocyte injury in OA by regulating EP300 mRNA stability.

Based on the above‐mentioned background, we hypothesized that YBX1 increases EP300 mRNA stability, which in turn elevates FOXC1 expression by promoting H3K27 acetylation at the FOXC1 promoter, ultimately aggravating chondrocyte injury and inflammatory responses during OA progression.

## 2 METHODS

### 2.1 Animal experiments

Male Sprague (standard deviation [SD]) rats (180–200 g) were purchased from Hunan Slike Jingda Co. Ltd. All experiments had received ethics approval from the Animal Ethics Committee of the Hainan General Hospital. Rats were housed under controlled conditions: 18–23°C, 50%–60% humidity, 12‐h light/dark cycle, and ad libitum food/water access. Twelve rats were randomized into a sham group (*n* = 6) and an anterior cruciate ligament transection (ACLT) group (*n* = 6). OA was induced via ACLT.^29^ Briefly, the rats were anesthetized with an intraperitoneal injection of 1% sodium pentobarbital (30 mg/kg). After anesthesia, the right hind limb and lower abdomen were shaved. A median incision over the anterior aspect of the right posterior knee exposes the joint. A 2‐cm curved incision along the medial patella allowed posterior rotation to visualize the joint cavity. The anterior cruciate ligament was transected with ophthalmic scissors, and instability was confirmed using a drawer test, which indicated a complete tear. The joint cavities were irrigated with saline and the incisions were closed with 4‐0 sutures. The rats resumed normal activities (eating/drinking) approximately 40 min postoperatively and were euthanized at 5 weeks for analysis.

### 2.2 Hematoxylin–eosin (H&E) and safranin O‐fast green (safranin O) staining

Tissue samples were fixed in 4% polyformaldehyde for 48 h, rinsed with tap water for 12 h, and decalcified in 12.5% EDTA for 8 weeks. After decalcification, samples were dehydrated using a series of ethanol gradients. Following paraffin embedding and sectioning, 5‐μm sagittal sections were stained with H&E (Solarbio) and safranin O (Solarbio).

### 2.3 Immunohistochemistry (IHC) assay

The knee joint tissue sections were dewaxed using Pro‐Par Clearant (Anatech Ltd.). The tissue was rehydrated using a descending ethanol gradient. Heat‐induced epitope retrieval was conducted in 10 mM sodium citrate buffer to expose antigenic sites. Nonspecific binding sites were blocked using normal goat serum prior to overnight incubation at 4°C with primary antibodies specific for YBX1 (PA5‐83493, Thermo), EP300 (ab61217, Abcam), and FOXC1 (ab223850, Abcam). Following incubation with the primary antibody, appropriate species‐matched secondary antibodies were used. Chromogenic detection was performed using the DAB substrate. Digital images of stained sections were acquired using a Nikon Eclipse optical imaging system.

### 2.4 Cell culture and treatment

Human chondrocyte cell line CHON‐001 was purchased from American Type Culture Collection and maintained in Dulbecco's modified Eagle medium (Thermo Fisher Scientific) supplemented with 10% fetal bovine serum (Gibco) and 1% penicillin and streptomycin (Beyotime) under the condition of 5% CO_2_ at 37°C. To build a cell model of OA, 10 ng/mL interleukin (IL)‐1β (Thermo Fisher Scientific) was used for intervening cells for 48 h. In addition, 20 μM C646 (MedChemExpress), an EP300 inhibitor, was used to incubate CHON‐001 cells for 24 h.

### 2.5 Cell transfection

Short hairpin RNA targeting FOXC1 (sh‐FOXC1), EP300 (sh‐EP300), and YBX1 (sh‐YBX1), overexpression plasmids including oe‐YBX1, oe‐EP300, and their corresponding negative controls, including sh‐NC and oe‐NC, were obtained from GenePharma. CHON‐001 cells were transfected with the plasmids for 48 h using Lipofectamine 3000 (Invitrogen).

### 2.6 Enzyme‐linked immunosorbent assay (ELISA)

The chondrocyte supernatant was collected after centrifugation. Then, referring to guideline documents, the concentrations of tumor necrosis factor (TNF)‐α and IL‐6 were determined by corresponding ELISA kits (Thermo Fisher Scientific).

### 2.7 Western blotting

Rat tissue samples and chondrocytes were lysed using a radio immunoprecipitation assay buffer (Beyotime) containing protease and phosphatase inhibitors (Beyotime). A bicinchoninic acid protein concentration determination kit (Beyotime) was used to measure the concentration of the extracted proteins, following the manufacturer's instructions. Equal quantities of protein (20 μg) were loaded into the loading holes of the gels and transferred onto polyvinylidene fluoride (PVDF) membranes. Skimmed milk (5%) was used to incubate PVDF membranes for 1 h. Afterward, primary antibodies of YBX1 (A303‐230A, 1:5000), FOXC1 (PA1‐807, 1:500), EP300 (MA1‐16622, 1:500), Bax (MA5‐14003, 1:50), caspase‐3 (43–7800, 1:250), Bcl‐2 (13–8800, 1:1000), collagen II (PA5‐99159, 1:2000), aggrecan (MA3‐16888, 1:1000), and GAPDH (MA5‐15738, 1:5000), purchased from Thermo Fisher Scientific, and β‐actin (ab8226, 1:2000) purchased from Abcam, were used for incubating PVDF membranes overnight at 4°C. The next day, the corresponding horseradish peroxidase‐conjugated secondary antibodies were incubated for 2 h. Subsequently, electrochemiluminescence reagent (Thermo Fisher Scientific) was used to visualize the protein bands. The gray values of the bands were determined using ImageJ software.

### 2.8 Flow cytometry

The apoptosis rate of chondrocytes was determined using flow cytometry. First, the chondrocytes subjected to different treatments were cultured and harvested. After washing with phosphate buffered saline, cells were resuspended in 100 μL of 1× binding buffer and then stained with a 10 μg/mL Annexin V/5 μL propidium iodide double‐staining solution (Dojindo) for 15 min at 37°C. Finally, cell apoptosis was analyzed using flow cytometry (Thermo Fisher Scientific).

### 2.9 Chromatin immunoprecipitation (ChIP)

Chondrocytes were cross‐linked with 1% formaldehyde, and chromatin was subsequently sonicated to generate fragments of approximately 200–1000 bp. Following centrifugation, the cell supernatant was incubated overnight at 4°C with the primary antibody against H3K27Ac (ab4729, Abcam) or IgG (ab172730, Abcam). Endogenous DNA‐protein complexes were then precipitated using protein A/G magnetic beads. After centrifugation, the precipitated complexes were washed, and immunoprecipitated DNA was analyzed using polymerase chain reaction (PCR).

### 2.10 RNA immunoprecipitation (RIP)

The interaction between YBX1 and EP300 mRNA was examined using the Magna RIP RBP Immunoprecipitation Kit (Millipore). Briefly, chondrocytes were harvested and lysed using the RIP lysis buffer. After centrifugation, cell lysates were incubated with anti‐YBX1 (A303‐230A; Thermo Fisher Scientific) and anti‐IgG (ab172730; Abcam) antibodies. Lysates were then supplemented with beads containing proteinase K, and RNA was isolated using the TRIzol reagent for immunoprecipitation. EP300 mRNA enrichment was measured by real‐time quantitative PCR (RT‐qPCR).

### 2.11 RNA pull‐down assay

Biotin‐labeled EP300 (Sangon) was transfected into chondrocytes. Following transfection, the chondrocytes were lysed, and the lysate was incubated with streptavidin‐coated magnetic beads to capture biotin‐labeled RNA complexes. Subsequently, the bound RNAs were isolated from the bead‐bound RNA complexes and YBX1 enriched with biotin‐labeled EP300 was detected by western blotting.

### 2.12 RT‐qPCR

Total RNA was extracted from chondrocytes using TRIzol reagent (Beyotime). Subsequently, cDNA was synthesized using the Script Reverse Transcription Reagent Kit (Takara). RT‐qPCR was performed using SYBR Premix Ex Taq (Takara) on the ABI Prism 7500 RT PCR System (Applied Biosystems). The classical 2^−ΔΔCT^ method was used to calculate the relative expression levels of the target genes, and the relative expression of genes was calculated based on the reference gene GAPDH. The primer sequences used were as follows:

YBX1 (F): 5′‐GCGGGGACAAGAAGGTCATC‐3′;
YBX1 (R): 5′‐CGAAGGTACTTCCTGGGGTTA‐3′;
EP300 (F): 5′‐AGCCAAGCGGCCTAAACTC‐3′;
EP300 (R): 5′‐TCACCACCATTGGTTAGTCCC‐3′;
GAPDH forward: 5′‐GGAGCGAGATCCCTCCAAAAT‐3′;
GAPDH reverse: 5′‐GGCTGTTGTCATACTTCTCATGG‐3′.

### 2.13 Stability of EP300 mRNA

Chondrocytes transfected with sh‐YBX1 or oe‐YBX1 were treated with 5 mg/mL actinomycin D, which was used to stop mRNA synthesis, for 0, 3, 6, and 9 h. Next, the cells were collected, RNA was extracted, and RT‐qPCR was used to examine EP300 mRNA levels.

### 2.14 Data analysis

All data were analyzed using GraphPad Prism 9, are shown as mean ± SD, and satisfied a normal distribution. For statistical comparisons, Student's *t*‐test was used to assess the differences between two independent groups. In cases involving multiple groups, one‐way analysis of variance was first performed to evaluate the overall significance, followed by Tukey's post‐hoc test for comparisons to identify specific group differences. Statistical significance was set at *p* < 0.05. To ensure reliability and reproducibility, all experimental data were obtained from at least three independent replicates.

## 3 RESULTS

### 3.1 YBX1, EP300, and FOXC1 expression was upregulated in ACLT model rats

**FIGURE 1:**
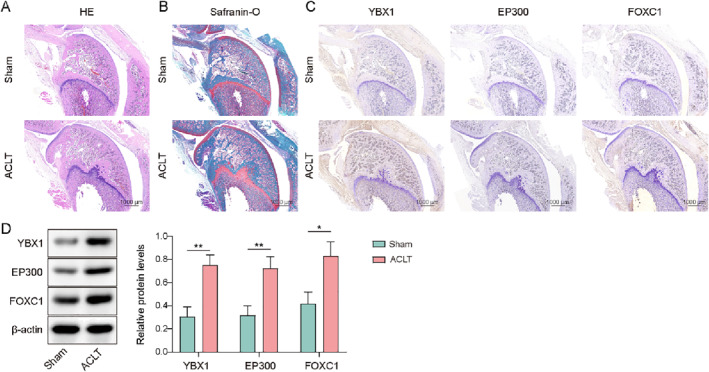
Y‐box binding protein 1 (YBX1), EP300, and forkhead box C1 (FOXC1) expression were upregulated in anterior cruciate ligament transection (ACLT) model rats. The rats were subjected to ACLT. (A, B) Pathological conditions of cartilage were evaluated using H&E and safranin O staining (scale bar = 1000 μm). (C, D) YBX1, EP300, and FOXC1 levels were determined by IHC and western blotting (scale bar = 1000 μm). *p < 0.05, **p < 0.01. Student's *t*‐test for (D). *n* = 6.

A rat OA model was established using the ACLT method to detect the expression of YBX1, EP300, and FOXC1 in OA. Subsequently, cartilage pathology was assessed via H&E and safranin O staining, which revealed superficial cartilage fibrillation, disordered chondrocyte arrangement, cartilage layer thinning, and even fissures or defects, confirming the successful induction of the ACLT model (Figure 1A,B). Furthermore, YBX1, EP300, and FOXC1 levels were assessed using IHC and western blotting. The results demonstrated a significant upregulation of YBX1, EP300, and FOXC1 in the cartilage tissues of ACLT model rats (Figure 1C,D). Taken together, these results suggest that YBX1, EP300, and FOXC1 participate in the development of OA.

### 3.2 FOXC1 knockdown attenuated IL‐1β‐induced chondrocyte apoptosis and inflammatory response

**FIGURE 2:**
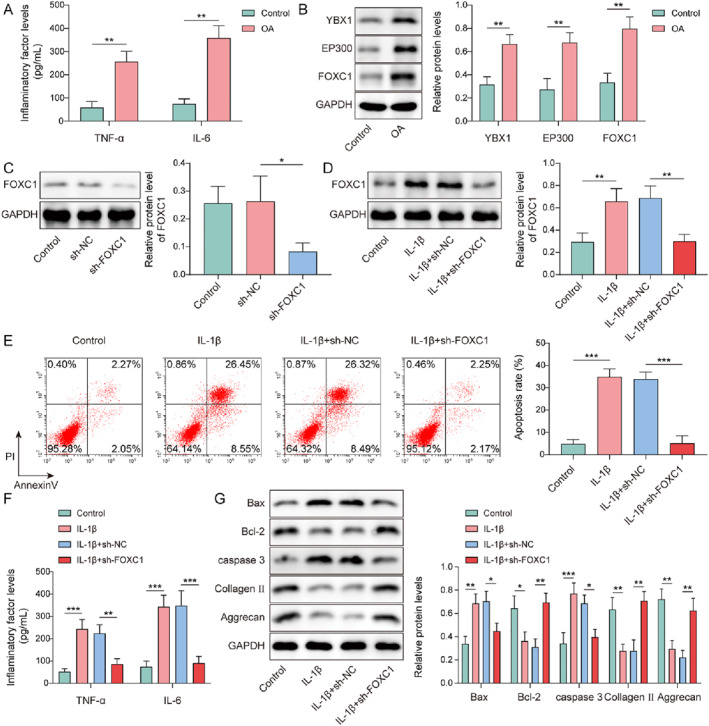
Forkhead box C1 (FOXC1) knockdown attenuated interleukin (IL)‐1β‐induced chondrocytes apoptosis and inflammatory response. Chondrocytes were treated with IL‐1β. (A) Tumor necrosis factor (TNF)‐α and IL‐6 levels were evaluated using enzyme‐linked immunosorbent assay (ELISA). (B) Y‐box binding protein 1 (YBX1), EP300, and FOXC1 expression levels were determined by western blotting. Chondrocytes were transfected with sh‐FOXC1 plasmids. (C) FOXC1 expression in sh‐FOXC1‐transfected chondrocytes was detected using western blotting. sh‐FOXC1‐transfected chondrocytes were treated with IL‐1β. (D) FOXC1 expression was evaluated using western blotting. (E) Apoptosis was assessed using flow cytometry. (F) TNF‐α and IL‐6 levels were evaluated using ELISA. (G) Bax, caspase‐3, Bcl‐2, collagen II, and aggrecan expression was determined by western blotting. *p < 0.05, **p < 0.01, ***p < 0.001. Student's *t*‐test for (A, B). One‐way analysis of variance followed by Tukey's post‐hoc test was used for (C–G). *n* = 3.

Chondrocytes were treated with IL‐1β to mimic chondrocyte injury during OA. TNF‐α and IL‐6 levels were greatly elevated in IL‐1β‐treated chondrocytes (Figure 2A). As previously described, YBX1, EP300 and FOXC1 have been implicated in OA progression.^12^, ^22^, ^27^ In the current study, the expression of YBX1, EP300, and FOXC1 was abnormally elevated in IL‐1β‐induced chondrocytes (Figure 2B). To further investigate the effect of FOXC1 on chondrocyte injury, sh‐FOXC1 plasmids were transfected into chondrocytes. sh‐FOXC1 exhibited efficient knockdown, as reflected by the significant decrease in FOXC1 expression after sh‐FOXCl transfection (Figure 2C). When sh‐FOXC1‐transfected chondrocytes were subjected to IL‐1β, IL‐1β‐mediated upregulation of FOXC1 was compromised by sh‐FOXC1 (Figure 2D). The apoptosis rate was raised in chondrocytes upon IL‐1β treatment, which was partially reversed by FOXC1 knockdown (Figure 2E). Besides, IL‐1β‐induced elevation of TNF‐α and IL‐6 levels in chondrocytes was offset by FOXC1 knockdown (Figure 2F). Furthermore, in IL‐1β‐induced chondrocytes, Bax, and caspase‐3 expression levels were elevated, whereas Bcl‐2, collagen II, and aggrecan expression levels were reduced. However, these changes were reversed by FOXC1 knockdown (Figure 2G). Therefore, these results demonstrated that FOXC1 knockdown could alleviate IL‐β‐induced chondrocyte apoptosis and inflammation.

### 3.3 YBX1 knockdown inhibited IL‐1β‐induced chondrocyte apoptosis and inflammatory response, which was aggravated by YBX1 overexpression

**FIGURE 3:**
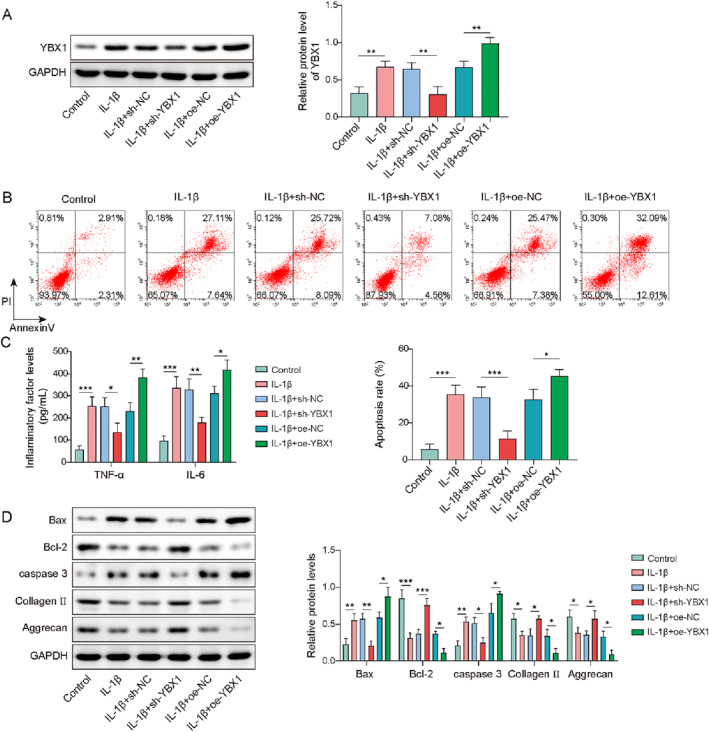
Y‐box binding protein 1 (YBX1) knockdown inhibited interleukin (IL)‐1β‐induced chondrocyte apoptosis and inflammatory response, which was aggravated by YBX1 overexpression. Chondrocytes were transfected with sh‐YBX1 or oe‐YBX1 followed by IL‐1β treatment. (A) YBX1 expression was detected using western blotting. (B) Apoptosis was assessed using flow cytometry. (C) Tumor necrosis factor (TNF)‐α and IL‐6 levels were evaluated using enzyme‐linked immunosorbent assay (ELISA). (D) Bax, caspase‐3, Bcl‐2, collagen II, and aggrecan expressions were determined by western blotting. *p < 0.05, **p < 0.01, ***p < 0.001. One‐way analysis of variance followed by Tukey's post‐hoc test was used for (A–D). *n* = 3.

To investigate the effects of YBX1 on chondrocytes in OA, firstly, chondrocytes were transfected with sh‐YBX1 or oe‐YBX1, followed by treatment with IL‐1β. IL‐1β treatment significantly upregulated the YBX1 expression in chondrocytes (Figure 3A). This upregulation was reversed by YBX1 knockdown but was further enhanced by YBX1 overexpression. Additionally, IL‐1β treatment led to increased chondrocyte apoptosis, elevated expression of the pro‐inflammatory cytokines TNF‐α and IL‐6, upregulation of Bax and caspase‐3, and downregulation of Bcl‐2, collagen II, and aggrecan. It is worth noting that YBX1 knockdown effectively reversed these changes, whereas YBX1 overexpression exacerbated the IL‐1β‐induced effects (Figure 3B–D). Overall, YBX1 silencing attenuated IL‐β‐induced apoptosis and inflammation in the cartilage, whereas YBX1 overexpression aggravated the changes.

### 3.4 EP300 promoted FOXC1 H3K27Ac in IL‐1β‐induced chondrocytes

**FIGURE 4:**
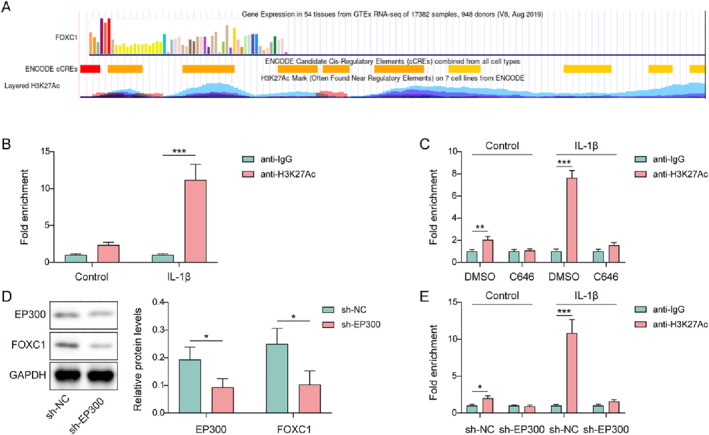
EP300 promoted forkhead box C1 (FOXC1) H3K27Ac in IL‐1β‐induced chondrocytes. (A) H3K27Ac enrichment in the FOXC1 promoter region was derived from ENCODE database, and visualized by the UCSC database. (B) H3K27Ac enrichment in the FOXC1 promoter in interleukin (IL)‐1β‐induced chondrocytes was validated using chromatin immunoprecipitation (ChIP). (C) H3K27Ac enrichment in the FOXC1 promoter was validated using ChIP in IL‐1β and C646‐treated chondrocytes. (D) The expression of EP300 and FOXC1 was evaluated in sh‐EP300‐transfected chondrocytes using western blotting. (E) H3K27Ac enrichment in the FOXC1 promoter was validated using ChIP in sh‐EP300‐transfected chondrocytes upon IL‐1β treatment. *p < 0.05, **p < 0.01, ***p < 0.001. Student's *t*‐test for (B–E). *n* = 3.

As shown in Figure 4A, H3K27Ac enrichment at the FOXC1 promoter was presented from the UCSC database (https://genome‐asia.ucsc.edu/), derived from datasets (ENCSR612NL, ENCSR532GIR, ENCSR447YYN, ENCSR977QPF) on the ENCODE website (https://www.encodeproject.org/). Furthermore, in IL‐1β‐induced chondrocytes, H3K27Ac enrichment in the FOXC1 promoter was observably enhanced, which was validated by ChIP (Figure 4B). However, after treatment with the EP300 inhibitor C646, H3K27Ac enrichment in the FOXC1 promoter was suppressed in IL‐1β‐induced chondrocytes (Figure 4C). Subsequently, sh‐EP300 was transfected with the chondrocytes. As presented in Figure 4D, sh‐EP300 transfection resulted in the downregulation of EP300 and FOXC1 levels. Similar to C646, EP300 knockdown suppressed H3K27Ac enrichment of the FOXC1 promoter in IL‐1β‐induced chondrocytes (Figure 4E). Collectively, FOXC1 H3K27Ac in IL‐1β‐induced chondrocytes was positively regulated by EP300.

### 3.5 EP300 upregulated FOXC1 to trigger IL‐1β‐induced chondrocytes apoptosis and inflammatory response

**FIGURE 5:**
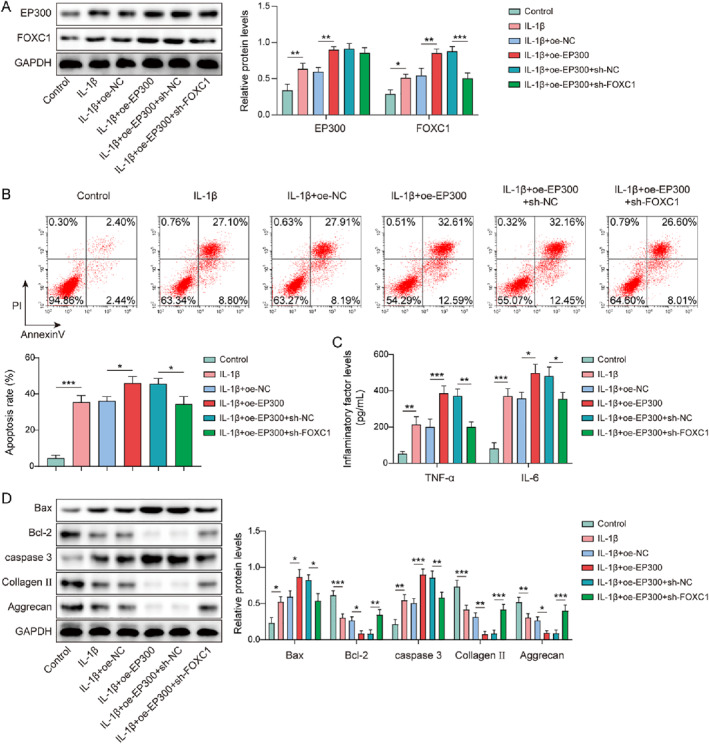
EP300 upregulated forkhead box C1 (FOXC1) to trigger interleukin (IL)‐1β‐induced chondrocytes apoptosis and inflammatory response. Chondrocytes were transfected with oe‐EP300 or in combination with sh‐FOXC1 and then followed by IL‐1β treatment. (A) EP300 and FOXC1 expression levels were determined by western blotting. (B) Apoptosis was assessed using flow cytometry. (C) Tumor necrosis factor (TNF)‐α and IL‐6 levels were evaluated using enzyme‐linked immunosorbent assay (ELISA). (D) Bax, caspase‐3, Bcl‐2, collagen II, and aggrecan expressions were determined by western blotting. *p < 0.05, **p < 0.01, ***p < 0.001. One‐way analysis of variance (ANOVA) followed by Tukey's post‐hoc test was used for (A–D). *n* = 3.

In the present study, we investigated the influence of the EP300/FOXC1 axis on chondrocyte injury in OA. Firstly, chondrocytes were transfected with oe‐EP300 or sh‐FOXC1 and then treated with IL‐1β. IL‐1β‐induced elevation of EP300 and FOXC1 expression was strengthened by EP300 overexpression; however, FOXC1 knockdown could weaken the influences of EP300 overexpression (Figure 5A). Furthermore, EP300 overexpression further exacerbated IL‐1β‐induced cell apoptosis, which was compromised by FOXC1 knockdown (Figure 5B). In addition, IL‐1β elevated TNF‐α and IL‐6 levels in chondrocytes and this effect was further potentiated by EP300 overexpression. However, FOXC1 knockdown attenuated EP300 overexpression‐induced TNF‐α and IL‐6 levels in IL‐1β‐treated chondrocytes (Figure 5C). Moreover, EP300 overexpression enhanced the expression of Bax and caspase‐3 while reducing the expression of Bcl‐2, collagen II, and aggrecan in IL‐1β‐induced chondrocytes, whereas FOXC1 knockdown impaired these influences (Figure 5D). Taken together, EP300 promoted IL‐1β‐induced chondrocyte apoptosis and inflammatory responses by upregulating FOXC1.

### 3.6 YBX1 enhanced the stability of EP300 mRNA

**FIGURE 6:**
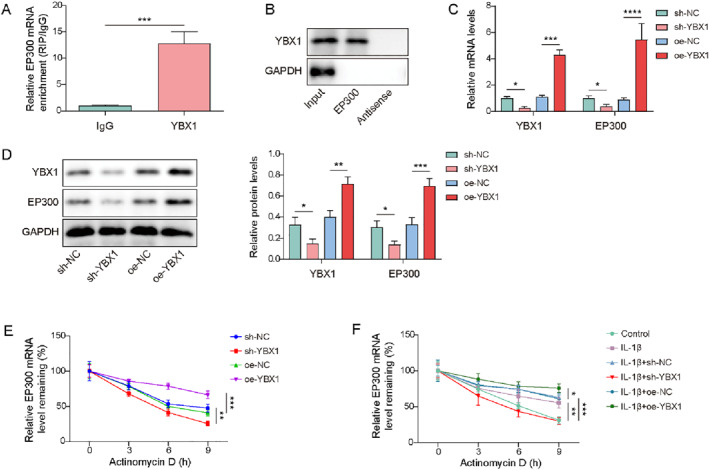
Y‐box binding protein 1 (YBX1) enhanced the stability of EP300 mRNA. (A, B) The interaction between YBX1 and EP300 mRNA was validated using RNA immunoprecipitation (RIP) and RNA pull‐down. (C, D) The expression of EP300 and YBX1 was detected in chondrocytes transfected with sh‐YBX1 or oe‐YBX1 using real‐time quantitative PCR (RT‐qPCR) and western blotting. (E) The stability of EP300 mRNA in sh‐YBX1 or oe‐YBX1‐transfected chondrocytes after actinomycin D treatment was evaluated using RT‐qPCR. (F) The stability of EP300 mRNA in sh‐YBX1 or oe‐YBX1‐transfected interleukin (IL)‐1β‐induced chondrocytes after actinomycin D treatment was evaluated by RT‐qPCR. *p < 0.05, **p < 0.01, ***p < 0.001. Student's *t*‐test for (A). One‐way analysis of variance (ANOVA) followed by Tukey's post‐hoc test for (B–F). *n* = 3.

YBX1 is an RBP that stabilizes the target gene mRNAs by interacting with mRNA transcripts.^30^ Using RIP and RNA pull‐down assay, we validated the interaction between YBX1 and EP300 mRNA, as the YBX1 antibody successfully enriched EP300 mRNA, and EP300 mRNA was pulled down by YBX1 protein (Figure 6A,B). YBX1 knockdown led to a reduction in YBX1 and EP300 expression, whereas YBX1 overexpression had the opposite effect (Figure 6C,D). Chondrocytes were treated with actinomycin D for different times (3, 6, 9 h) and EP300 mRNA was detected. The results displayed that YBX1 knockdown attenuated the EP300 mRNA stability, whereas YBX1 overexpression enhanced it (Figure 6E). Besides, IL‐1β enhanced the stability of EP300 mRNA. This stabilization was abolished by YBX1 knockdown but was further promoted by YBX1 overexpression (Figure 6F). In summary, YBX1 stabilized EP300 mRNA by binding to EP300 in chondrocytes.

### 3.7 YBX1 activated the EP300/FOXC1 axis to promote IL‐1β‐induced chondrocytes apoptosis and inflammation

**FIGURE 7:**
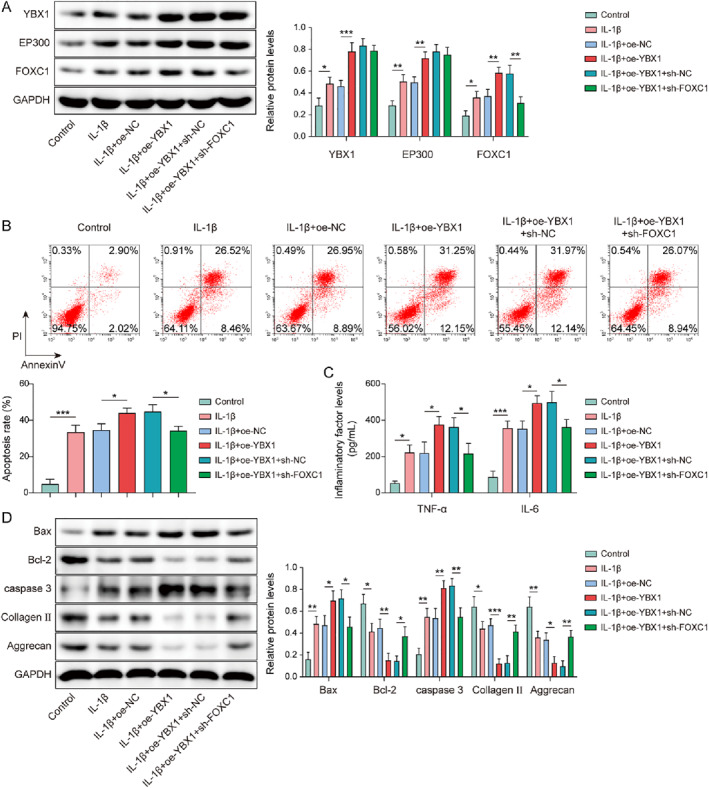
Y‐box binding protein 1 (YBX1) activated the EP300/forkhead box C1 (FOXC1) axis to promote IL‐1β‐induced chondrocytes apoptosis and inflammation. Chondrocytes were transfected with oe‐YBX1 alone or in combination with sh‐FOXC1. (A) YBX1, EP300, and FOXC1 expression levels were determined by western blotting. (B) Apoptosis was assessed using flow cytometry. (C) Tumor necrosis factor (TNF)‐α and IL‐6 levels were evaluated using enzyme‐linked immunosorbent assay. (D) Bax, caspase‐3, Bcl‐2, collagen II, and aggrecan expression was determined by western blotting. *p < 0.05, **p < 0.01, ***p < 0.001. One‐way analysis of variance followed by Tukey's post‐hoc test was used for (A–D). *n* = 3.

To investigate the role of the YBX1/EP300/FOXC1 axis in chondrocyte injury, IL‐1β‐induced chondrocytes were transfected with oe‐YBX1 or sh‐FOXC1. YBX1 overexpression further increased the YBX1, EP300, and FOXC1 levels in IL‐1β‐induced chondrocytes. It is worth noting that FOXC1 knockdown selectively reversed YBX1 overexpression‐mediated FOXC1 upregulation without affecting YBX1 or EP300 expression (Figure 7A). YBX1 upregulation exacerbated apoptosis in IL‐1β‐induced chondrocytes, whereas FOXC1 knockdown eliminated this effect (Figure 7B). Additionally, YBX1 overexpression enhanced IL‐1β‐mediated upregulation of TNF‐α and IL‐6 levels, which was offset by FOXC1 knockdown (Figure 7C). Furthermore, YBX1 overexpression elevated the expression of Bax and caspase‐3 while decreasing the expression of Bcl‐2, collagen II, and aggrecan in IL‐1β‐treated chondrocytes, which was impaired by FOXC1 inhibition (Figure 7D). Collectively, these results suggested that YBX1 promoted IL‐1β‐treated chondrocyte injury and inflammation by regulating the EP300/FOXC1 axis.

Furthermore, YBX1 overexpression increased Bax and caspase‐3 while decreasing Bcl‐2, collagen II, and aggrecan in IL‐1β‐treated chondrocytes; these changes were negated by FOXC1 inhibition (Figure 7D). Collectively, these results indicate that YBX1 promotes IL‐1β‐induced chondrocyte injury and inflammation by regulating the EP300/FOXC1 axis.

## 4 DISCUSSION

OA is a chronic joint disease characterized by the pathological degeneration of articular cartilage.^28^ Articular cartilage consists of chondrocytes and extracellular matrix (ECM) synthesized and secreted by chondrocytes; abnormal ECM degradation and disrupted homeostasis lead to articular cartilage degeneration. The ECM serves as an external scaffold for chondrocytes.^31^ It is worth noting that normal cartilage ECM is primarily composed of collagen II and aggrecan. Signal transduction, which governs chondrocyte growth, differentiation, and apoptosis, is closely linked to the ECM.^32^ Therefore, it is crucial to explore the pathological mechanisms underlying OA chondrocyte injury and inflammation is essential. In OA research, the selection of animal and cell models is of utmost importance. The ACLT animal model can effectively simulate OA induced by mechanical injury.^33^ Additionally, as a key inflammatory cytokine in OA, IL‐1β could be used to induce chondrocytes for constructing an in vitro cartilage injury model of OA.^34^ In this study, we employed ACLT surgery to establish a rat OA model and IL‐1β‐induced chondrocytes to construct an in vitro OA model, respectively. Our findings revealed that YBX1, EP300 and FOXC1 were observably elevated in ACLT rats and IL‐1β‐induced chondrocytes. Additionally, YBX1 could stabilize EP300 mRNA to elevate EP300 expression, which regulated H3K27Ac enrichment in the FOXC1 promoter, and then promoted IL‐1β‐induced cell apoptosis and inflammatory response in chondrocytes.

FOXC1 is a well‐studied transcription factor. Accumulating evidence suggests that it plays a significant role in various diseases, including OA.^12^ Few studies have clarified how FOXC1 is involved in OA progression. For instance, FOXC1 expression was upregulated in IL‐1β‐induced synovial fibroblasts, which was closely associated with OA progression, and FOXC1 silencing attenuated inflammatory responses in these cells.^11^ Of note, a published study reported that FOXC1 overexpression abolished miR‐138‐5p overexpression‐mediated promotion of ECM degradation in IL‐1β‐induced chondrocytes,^35^ indicating that FOXC1 acts as a protector in chondrocyte injury in OA. However, in our study, we found that FOXC1 expression was elevated in ACLT rats and IL‐1β‐induced chondrocytes. Besides, IL‐1β treatment promoted inflammatory responses, cell apoptosis, and ECM degradation, whereas FOXC1 knockdown attenuated IL‐1β‐induced alterations. Our results suggested that FOXC1 may act as a promotes cartilage damage in OA, contrary to the findings reported in previously published studies.^35^, ^36^ Studies supporting our results have been published.^12^, ^37^, ^38^ Research on the mechanism of action of FOXC1 in OA cartilage injury remains relatively limited. The discrepancy between the results of this study and those of some published studies may be attributed to the following reasons. First, heterogeneity in the research design. Differences in cell types used across studies and cartilage injury induction conditions (such as induction by IL‐1β or lipopolysaccharide (LPS), concentration, and intervention duration) may lead to different regulatory patterns of FOXC1 function. Second, there were limitations to in vitro and in vivo studies. Existing conclusions are mostly based on in vitro cell experiments and lack sufficient validation in animal models, and the influence of the complex in vivo microenvironment on FOXC1 function remains unclear. Third, the sample size and the experimental reproducibility were insufficient. The small sample sizes or low repetition times in some studies may have reduced the reproducibility of the conclusions and affected the reliability of the results. These factors collectively contribute to the unclear specific mechanism of action of FOXC1 in OA, which requires further systematic clarification through multi‐model validation, large‐sample analysis, and integrated in vitro and in vivo research.

Protein acetylation is an important regulatory mechanism by which cells control gene expression, protein activity, and physiological processes.^39^ Enhanced FOXC1 expression in breast cancer cells is associated with increased acetylation of histone H3, which is regulated by O‐GlcNAc transferase depletion.^40^ In addition, PcG proteins mediate the elevation of FOXC1 expression via H3/H4 acetylation of the FOXC1 promoter in breast cancer.^16^ Therefore, highly abnormal FOXC1 expression in IL‐1β‐induced chondrocytes may be related to the acetylation of the FOXC1 promoter. Using the ENCODE project at UCSC website, we identified H3K27Ac deposition at the FOXC1 promoter region. This finding is confirmed in the present study. Our experimental evidence validated higher H3K27Ac at the FOXC1 promoter in IL‐1β‐induced chondrocytes. Increasing evidence indicates that EP300 is a vital histone acetyltransferase that mediates H3K27Ac modification. For example, JQAD1, an EP300 degrader, reduced H3K27Ac levels in transcriptional core regulatory circuitry enhancers.^41^ Dang Wei et al. reported that EP300‐mediated H3K27Ac of the KIF11 promoter enhanced KIF11 expression in gallbladder cancer.^42^ In the present study, we found that silencing C646, an inhibitor of EP300, or EP300 reduced H3K27Ac levels at the FOXC1 promoter in IL‐1β‐induced chondrocytes, whereas EP300 overexpression produced opposite results. Moreover, EP300 downregulation reduced FOXC1 expression. The above results validated that EP300 mediated H3K27Ac of FOXC1 promoter to elevate FOXC1 in IL‐1β‐induced chondrocytes. It is worth noting that EP300 has been reported to correlate with OA progression. Network pharmacology and molecular docking technologies have suggested that EP300 is a core target of various OA therapeutic drugs.^43^ Additionally, EP300, a spermidine target, affects autophagy and polyamine synthesis in osteoarthritic chondrocytes.^22^ In this study, we found that EP300 overexpression further exacerbated the influences of IL‐1β treatment on chondrocyte apoptosis and inflammatory responses. As expected, the FOXC1 knockdown reversed the effects of EP300 overexpression. Taken together, EP300 promotes chondrocyte injury and inflammation by mediating H3K27Ac enrichment in the FOXC1 promoter.

YBX1 is a common RBP containing a cold shock domain that enables specific RNA binding and influences the stability of target gene mRNA.^44^, ^45^ For example, YBX1 interacts with SOX2 mRNA to enhance its stability in esophageal squamous cell carcinoma.^30^ Using the StarBase database, we identified a predicted YBX1 binding site on EP300 mRNA that was experimentally validated in our study. YBX1 has also been implicated in OA progression. For examples, miR‐379‐5p silences YBX1 to promote chondrocyte proliferation in OA.^27^ Here, we observed aberrant upregulation of YBX1 in ACLT rats and IL‐1β‐induced chondrocytes, and YBX1 overexpression exacerbated IL‐1β‐induced chondrocyte injury. Furthermore, YBX1 overexpression increased FOXC1 expression. As expected, FOXC1 knockdown reversed YBX1 overexpression‐mediated exacerbation of chondrocyte injury and inflammation.

In conclusion, our findings suggested that YBX1 stabilizes EP300 mRNA, thereby upregulating EP300 expression and enhancing H3K27Ac enrichment in the FOXC1 promoter through EP300‐mediated acetylation. This results in elevated FOXC1 expression, which subsequently exacerbates chondrocyte injury and the inflammatory response in OA. These insights may pave the way for the identification of potential therapeutic targets in OA in the future.

However, this study had certain limitations. First, the current conclusions were primarily derived from experiments using IL‐1β to culture cells. However, in OA, there are interactions between multiple substances, and the experimental environment differs from the actual situation, which may lead to incomplete results. Second, in the rat surgical model experiments, we did not perform gain‐of‐function or loss‐of‐function experiments on specific genes; therefore, the true causal relationship between the YBX1‐EP300‐FOXC1 signaling pathway and OA could not be confirmed. Third, how YBX1 is activated and which downstream targets of FOXC1 are affected remain unclear, and these upstream and downstream regulatory relationships require further exploration. Finally, in studies on other diseases, FOXC1 was shown to be regulated by various epigenetic modifications such as ubiquitination, DNA methylation, and SUMOylation.^46^, ^47^, ^48^ Besides, USP15 positively regulate the expression of FOXC1 by regulating its deubiquitination to induce M2 polarization of macrophages, thereby alleviating LPS‐induced chondrocyte injury in OA.^36^ Future studies should explore the role of FOXC1 in OA from the perspective of epigenetic modifications. Therefore, in the future, researchers should attempt to use more diverse experimental models, combine gene intervention experiments with cutting‐edge analytical techniques, and conduct in‐depth investigations into the limitations of this study.

## AUTHOR CONTRIBUTIONS

**Jingyi Li**: Conceptualization; writing—original draft; project administration; funding acquisition. **Gang Zhou**: Validation; investigation; resources. **Te Chen**: Supervision; methodology. **Qiao Lin**: Formal analysis; data curation. **Qiupeng Yang**: Visualization; writing—review and editing.

## CONFLICT OF INTEREST STATEMENT

The authors declare no conflicts of interest.

## ETHICS STATEMENT

All animal experiments had received ethics approval from the Animal Ethics Committee of the Hainan General Hospital.

## Data Availability

The raw data supporting the conclusions of this manuscript will be made available by the authors, without undue reservation, to any qualified researcher.

## References

[1] Liu, Suqing , Yurong Pan , Ting Li , Mi Zou , Wenji Liu , Qingqing Li , Huan Wan , Jie Peng , and Liang Hao . 2023. “The Role of Regulated Programmed Cell Death in Osteoarthritis: From Pathogenesis to Therapy.” International Journal of Molecular Sciences 24(6): 5364. 10.3390/ijms24065364. 36982438 PMC10049357

[2] Katz, Jeffrey N. , Kaetlyn R. Arant , and Richard F. Loeser . 2021. “Diagnosis and Treatment of Hip and Knee Osteoarthritis: A Review.” JAMA 325(6): 568–578. 10.1001/jama.2020.22171. 33560326 PMC8225295

[3] Pincus, Daniel , Richard Jenkinson , Michael Paterson , Timothy Leroux , and Bheeshma Ravi . 2020. “Association between Surgical Approach and Major Surgical Complications in Patients Undergoing Total Hip Arthroplasty.” JAMA 323(11): 1070–1076. 10.1001/jama.2020.0785. 32181847 PMC7078797

[4] Qiu, Huanhuan , Wei Wang , Kejun Hu , Wangwang Liu , Shumin Pan , Qi Lv , Guanglin Xu , and Qingfeng Yu . 2023. “EuHD1 Protects against Inflammatory Injury Driven by NLRP3 Inflammasome.” International Immunopharmacology 115: 109712. 10.1016/j.intimp.2023.109712. 37724954

[5] Zamli, Zaitunnatakhin , Michael Adams , John Tarlton , and Mohammed Sharif . 2013. “Increased Chondrocyte Apoptosis Is Associated with Progression of Osteoarthritis in Spontaneous Guinea Pig Models of the Disease.” International Journal of Molecular Sciences 14(9): 17729–17743. 10.3390/ijms140917729. 23994836 PMC3794750

[6] Katoh, Masuko , Maki Igarashi , Hirokazu Fukuda , Hitoshi Nakagama , and Masaru Katoh . 2013. “Cancer Genetics and Genomics of Human FOX Family Genes.” Cancer Letters 328(2): 198–206. 10.1016/j.canlet.2012.09.017. 23022474

[7] He, Tailin , Jialin Shang , Chenglong Gao , Xin Guan , Yingyi Chen , Liwen Zhu , Luyong Zhang , Cunjin Zhang , Jian Zhang , and Tao Pang . 2021. “A Novel SIRT6 Activator Ameliorates Neuroinflammation and Ischemic Brain Injury via EZH2/FOXC1 Axis.” Acta Pharmaceutica Sinica B 11(3): 708–726. 10.1016/j.apsb.2020.11.002. 33777677 PMC7982432

[8] Oraby, Mamdouh A. , Sherif S. Abdel Mageed , Ahmed Amr Raouf , Dareen A. Abdelshafy , Eman F. Ahmed , Rowida T. Khalil , Safwat A. Mangoura , and Doaa S. Fadaly . 2024. “Remdesivir Ameliorates Ulcerative Colitis‐Propelled Cell Inflammation and Pyroptosis in Acetic Acid Rats by Restoring SIRT6/FoxC1 Pathway.” International Immunopharmacology 137: 112465. 10.1016/j.intimp.2024.112465. 38878489

[9] Wei, Yazhi , Xinmin Huang , Yanmei Ma , and Liping Dai . 2022. “FOXC1‐mediated TRIM22 Regulates the Excessive Proliferation and Inflammation of Fibroblast‐like Synoviocytes in Rheumatoid Arthritis via NF‐κB Signaling Pathway.” Molecular Medicine Reports 26(4): 304. 10.3892/mmr.2022.12820. 35946462 PMC9434987

[10] Fei, Qi , JiSheng Lin , Hai Meng , BingQiang Wang , Yong Yang , Qi Wang , Nan Su , Jinjun Li , and Dong Li . 2016. “Identification of Upstream Regulators for Synovial Expression Signature Genes in Osteoarthritis.” Joint Bone Spine 83(5): 545–551. 10.1016/j.jbspin.2015.09.001. 26832188

[11] He, Xiao , and Lili Deng . 2022. “miR‐204‐5p Inhibits Inflammation of Synovial Fibroblasts in Osteoarthritis by Suppressing FOXC1.” Journal of Orthopaedic Science 27(4): 921–928. 10.1016/j.jos.2021.03.014. 34045139

[12] Wang, Jun , Yin Wang , Hui Zhang , Weilu Gao , Ming Lu , Wendong Liu , Yetian Li , and Zongsheng Yin . 2020. “Forkhead Box C1 Promotes the Pathology of Osteoarthritis by Upregulating β‐Catenin in Synovial Fibroblasts.” FEBS Journal 287(14): 3065–3087. 10.1111/febs.15178. 31837247

[13] Shvedunova, Maria , and Asifa Akhtar . 2022. “Modulation of Cellular Processes by Histone and Non‐Histone Protein Acetylation.” Nature Reviews Molecular Cell Biology 23(5): 329–349. 10.1038/s41580-021-00441-y. 35042977

[14] Zaib, Sumera , Nehal Rana , and Imtiaz Khan . 2022. “Histone Modifications and Their Role in Epigenetics of Cancer.” Current Medicinal Chemistry 29(14): 2399–2411. 10.2174/0929867328666211108105214. 34749606

[15] Núñez‐Carro, Carmen , Margarita Blanco‐Blanco , Karla Mariuxi Villagrán‐Andrade , Francisco J. Blanco , and María C. de Andrés . 2023. “Epigenetics as a Therapeutic Target in Osteoarthritis.” Pharmaceuticals (Basel) 16(2): 156. 10.3390/ph16020156. 37259307 PMC9964205

[16] Du, Juan , Lin Li , Zhouluo Ou , Chenfei Kong , Yu Zhang , Zhixiong Dong , Shan Zhu , et al. 2012. “FOXC1, a Target of Polycomb, Inhibits Metastasis of Breast Cancer Cells.” Breast Cancer Research and Treatment 131(1): 65–73. 10.1007/s10549-011-1396-3. 21465172

[17] Zhang, Bing , Daniel S. Day , Joshua W. Ho , Lingyun Song , Jingjing Cao , Danos Christodoulou , Jonathan G. Seidman , Gregory E. Crawford , Peter J. Park , and William T. Pu . 2013. “A Dynamic H3K27ac Signature Identifies VEGFA‐Stimulated Endothelial Enhancers and Requires EP300 Activity.” Genome Research 23(6): 917–927. 10.1101/gr.149674.112. 23547170 PMC3668360

[18] Liu, Shan , Jie Zhou , Xiangling Ye , Danni Chen , Weimin Chen , Yaobin Lin , Zhizhong Chen , Biyun Chen , and Jin Shang . 2023. “A Novel lncRNA SNHG29 Regulates EP300‐ Related Histone Acetylation Modification and Inhibits FLT3‐ITD AML Development.” Leukemia 37(7): 1421–1434. 10.1038/s41375-023-01923-y. 37157016

[19] Magni, Martina , Giacomo Buscemi , Lucia Maita , Lei Peng , Siu Yuen Chan , Alessandra Montecucco , Domenico Delia , and Laura Zannini . 2019. “TSPYL2 Is a Novel Regulator of SIRT1 and p300 Activity in Response to DNA Damage.” Cell Death & Differentiation 26(5): 918–931. 10.1038/s41418-018-0168-6. 30050056 PMC6461906

[20] Bugyei‐Twum, Antoinette , Andrew Advani , Suzanne L. Advani , Yuan Zhang , Kerri Thai , Darren J. Kelly , and Kim A. Connelly . 2014. “High Glucose Induces Smad Activation via the Transcriptional Coregulator p300 and Contributes to Cardiac Fibrosis and Hypertrophy.” Cardiovascular Diabetology 13(1): 89. 10.1186/1475-2840-13-89. 24886336 PMC4108062

[21] Di Pietrantonio, Nadia , Pamela Di Tomo , Domitilla Mandatori , Gloria Formoso , and Assunta Pandolfi . 2023. “Diabetes and Its Cardiovascular Complications: Potential Role of the Acetyltransferase p300.” Cells 12(3): 431. 10.3390/cells12030431. 36766773 PMC9914144

[22] Sacitharan, Pradeep K. , Seint Lwin , George Bou Gharios , and James R. Edwards . 2018. “Spermidine Restores Dysregulated Autophagy and Polyamine Synthesis in Aged and Osteoarthritic Chondrocytes via EP300.” Experimental & Molecular Medicine 50(9): 1–10. 10.1038/s12276-018-0149-3. PMC6145946 30232322

[23] Velasco, Javier , M. T. Zarrabeitia , J. R. Prieto , Jose L. Perez‐Castrillon , Maria Dolores Perez‐Aguilar , Maria I. Perez‐Nuñez , Carolina Sañudo , et al. 2010. “Wnt Pathway Genes in Osteoporosis and Osteoarthritis: Differential Expression and Genetic Association Study.” Osteoporosis International 21(1): 109–118. 10.1007/s00198-009-0931-0. 19373426

[24] Feng, Mengdie , Xueqin Xie , Guoqiang Han , Tiantian Zhang , Yashu Li , Yicun Li , Rong Yin , et al. 2021. “YBX1 Is Required for Maintaining Myeloid Leukemia Cell Survival by Regulating BCL2 Stability in an m6A‐dependent Manner.” Blood 138(1): 71–85. 10.1182/blood.2020009676. 33763698 PMC8667054

[25] Hao, Linlin , Jian Zhang , Zhongshan Liu , Zhiliang Zhang , Tiezhu Mao , Jie Guo . 2023. “Role of the RNA‐Binding Protein Family in Gynecologic Cancers.” American Journal of Cancer Research 13(8): 3799–3821. 37693158 PMC10492115

[26] Lu, Xin , Shouqian Dai , Pengfei Li , Yuqian Zhou , and Feng Xu . 2024. “YBX‐1 Alleviates Sepsis‐Stimulated Lung Epithelial Cell Injury.” Allergologia et Immunopathologia 52(2): 60–67. 10.15586/aei.v52i2.1068. 38459892

[27] Zhang, Hongjun , Wendi Zheng , Du Li , and Jia Zheng . 2022. “MiR‐379‐5p Promotes Chondrocyte Proliferation via Inhibition of PI3K/Akt Pathway by Targeting YBX1 in Osteoarthritis.” Cartilage 13(1): 19476035221074024. 10.1177/19476035221074024. 35255737 PMC9137300

[28] Wu, Ruifan , Shuting Cao , Fan Li , Shengchun Feng , Gang Shu , Lina Wang , Ping Gao , et al. 2022. “RNA‐Binding Protein YBX1 Promotes Brown Adipogenesis and Thermogenesis via PINK1/PRKN‐Mediated Mitophagy.” FASEB Journal 36(3): e22219. 10.1096/fj.202101810rr. 35195911

[29] Yu, Shunan , Xiong Shu , Xinyu Wang , Yueyang Sheng , Shan Li , Ying Wang , Yanzhuo Zhang , Jiangfeng Tao , Xu Jiang , and Chengai Wu . 2024. “The Novel HSP90 Monoclonal Antibody 9B8 Ameliorates Articular Cartilage Degeneration by Inhibiting Glycolysis via the HIF‐1 Signaling Pathway.” Heliyon 10(16): e35603. 10.1016/j.heliyon.2024.e35603. 39229534 PMC11369415

[30] Lu, Jun‐Tao , Zhao‐Yang Yan , Tong‐Xin Xu , Fan Zhao , Lei Liu , Fei Li , and Wei Guo . 2023. “Reciprocal Regulation of LINC00941 and SOX2 Promotes Progression of Esophageal Squamous Cell Carcinoma.” Cell Death & Disease 14(1): 72. 10.1038/s41419-023-05605-6. 36717549 PMC9886991

[31] Wang, Gangliang , Shuai Chen , Ziang Xie , Shuying Shen , Wenbin Xu , Wenxiang Chen , Xiang Li , et al. 2020. “TGFβ Attenuates Cartilage Extracellular Matrix Degradation via Enhancing FBXO6‐Mediated MMP14 Ubiquitination.” Annals of the Rheumatic Diseases 79(8): 1111–1120. 10.1136/annrheumdis-2019-216911. 32409323 PMC7392491

[32] Musumeci, Giuseppe , Flavia Aiello , Marta Szychlinska , Michelino Di Rosa , Paola Castrogiovanni , and Ali Mobasheri . 2015. “Osteoarthritis in the XXIst Century: Risk Factors and Behaviours that Influence Disease Onset and Progression.” International Journal of Molecular Sciences 16(3): 6093–6112. 10.3390/ijms16036093. 25785564 PMC4394521

[33] Xie, Wenpeng , Shangfeng Qi , Luming Dou , Lei Wang , Xiangpeng Wang , Rongxiu Bi , Nianhu Li , and Yongkui Zhang . 2023. “Achyranthoside D Attenuates Chondrocyte Loss and Inflammation in Osteoarthritis via Targeted Regulation of Wnt3a.” Phytomedicine 111: 154663. 10.1016/j.phymed.2023.154663. 36657317

[34] Xiong, Jun , Wei Liu , Jianfei Chen , and Yi Niu . 2023. “Circ_0001721 Knockdown Relieves IL‐1β‐Induced Chondrocyte Injury via Regulating miR‐373‐3p/CXCR4 in Osteoarthritis.” International Immunopharmacology 115: 109455. 10.1016/j.intimp.2022.109455. 36608447

[35] Yuan, Y. , G. Q. Zhang , W. Chai , M. Ni , C. Xu , and J. Y. Chen . 2016. “Silencing of MicroRNA‐138‐5p Promotes IL‐1β‐Induced Cartilage Degradation in Human Chondrocytes by Targeting FOXC1: miR‐138 Promotes Cartilage Degradation.” Bone & Joint Research 5(10): 523–530. 10.1302/2046-3758.510.bjr-2016-0074.r2. 27799147 PMC5108353

[36] Liang, Qibin , Qinghe Ding , Liang Zhao , Jingchao Tan , and Wei Niu . 2025. “USP15‐modified ADMSCs‐Exo Alleviates Chondrocyte Damage and Effectively Relieved Osteoarthritis by Inducing M2 Polarization of Macrophages through Deubiquitinating FOXC1.” Journal of Orthopaedic Surgery and Research 20(1): 336. 10.1186/s13018-025-05742-y. 40176111 PMC11963356

[37] Li, Jingyi , Gang Zhou , Te Chen , Qiao Lin , and Qiupeng Yang . 2024. “LncRNA RMRP Promotes Chondrocyte Injury by Regulating the FOXC1/RBP4 Axis.” Central European Journal of Immunology 49(4): 366–382. 10.5114/ceji.2024.145312. 39944260 PMC11811726

[38] Li, Zhengyuan , Lin Hao , Shenghong Chen , Wenhan Fu , Hui Zhang , Zongsheng Yin , Yin Wang , and Jun Wang . 2024. “Forkhead Box C1 Promotes the Pathology of Osteoarthritis in Subchondral Bone Osteoblasts via the Piezo1/YAP Axis.” Cellular Signalling 124: 111463. 10.1016/j.cellsig.2024.111463. 39396563

[39] Wiesel‐Motiuk, Naama , and Yehuda G. Assaraf . 2020. “The Key Roles of the Lysine Acetyltransferases KAT6A and KAT6B in Physiology and Pathology.” Drug Resistance Updates 53: 100729. 10.1016/j.drup.2020.100729. 33130515

[40] Forma, Ewa , Paweł Jóźwiak , Piotr Ciesielski , Agnieszka Zaczek , Katarzyna Starska , Magdalena Bryś , and Anna Krześlak . 2018. “Impact of OGT Deregulation on EZH2 Target Genes FOXA1 and FOXC1 Expression in Breast Cancer Cells.” PLoS One 13(6): e0198351. 10.1371/journal.pone.0198351. 29864144 PMC5986130

[41] Durbin, Adam D. , Tingjian Wang , Virangika K. Wimalasena , Mark W. Zimmerman , Deyao Li , Neekesh V. Dharia , Luca Mariani , et al. 2022. “EP300 Selectively Controls the Enhancer Landscape of MYCN‐Amplified Neuroblastoma.” Cancer Discovery 12(3): 730–751. 10.1158/2159-8290.cd-21-0385. 34772733 PMC8904277

[42] Wei, Dang , Bian Rui , Fan Qingquan , Cai Chen , Hu Yun Ping , Song Xiaoling , Weng Hao , and Gu Jun . 2021. “KIF11 Promotes Cell Proliferation via ERBB2/PI3K/AKT Signaling Pathway in Gallbladder Cancer.” International Journal of Biological Sciences 17(2): 514–526. 10.7150/ijbs.54074. 33613109 PMC7893577

[43] Yang, Liu , Senwang Zheng , Ajiao Hou , Song Wang , Jiaxu Zhang , Huan Yu , Xuejiao Wang , and Wei Lan . 2022. “Discussion on the Molecular Mechanism of Duhuo Jisheng Decoction in Treating Osteoarthritis Based on Network Pharmacology and Molecular Docking.” Medicine (Baltimore) 101(42): e31009. 10.1097/md.0000000000031009. 36281111 PMC9592334

[44] Alkrekshi, Akram , Wei Wang , Priyanka Shailendra Rana , Vesna Markovic , and Khalid Sossey‐Alaoui . 2021. “A Comprehensive Review of the Functions of YB‐1 in Cancer Stemness, Metastasis and Drug Resistance.” Cellular Signalling 85: 110073. 10.1016/j.cellsig.2021.110073. 34224843 PMC8878385

[45] Mordovkina, Daria , Dmitry N. Lyabin , Egor A. Smolin , Ekaterina M. Sogorina , Lev P. Ovchinnikov , and Irina Eliseeva . 2020. “Y‐Box Binding Proteins in mRNP Assembly, Translation, and Stability Control.” Biomolecules 10(4): 591. 10.3390/biom10040591. 32290447 PMC7226217

[46] Danciu, Theodora E. , Sergey Chupreta , Osvaldo Cruz , Jennifer E. Fox , Malcolm Whitman , and Jorge A. Iñiguez‐Lluhí . 2012. “Small Ubiquitin‐Like Modifier (SUMO) Modification Mediates Function of the Inhibitory Domains of Developmental Regulators FOXC1 and FOXC2.” Journal of Biological Chemistry 287(22): 18318–18329. 10.1074/jbc.m112.339424. 22493429 PMC3365760

[47] Muggerud, Aslaug Aa , Jo Anders Rønneberg , Fredrik Wärnberg , Johan Botling , Florence Busato , Jovana Jovanovic , Hiroko Solvang , et al. 2010. “Frequent Aberrant DNA Methylation of ABCB1, FOXC1, PPP2R2B and PTEN in Ductal Carcinoma In Situ and Early Invasive Breast Cancer.” Breast Cancer Research 12(1): R3. 10.1186/bcr2466. 20056007 PMC2880421

[48] Wang, Jie , Lang Gan , Fenghao Liu , Qin Yang , Qingsong Deng , Di Jiang , Chengcheng Zhang , LeiDa Zhang , and XiaoJun Wang . 2024. “USP10 Promotes Pancreatic Ductal Adenocarcinoma Progression by Attenuating FOXC1 Protein Degradation to Activate the WNT Signaling Pathway.” International Journal of Biological Sciences 20(13): 5343–5362. 10.7150/ijbs.92278. 39430239 PMC11488585

